# Risk of Malignancy in Indeterminate Liver Nodules Among Patients with Cirrhosis: A Retrospective Cohort Study

**DOI:** 10.1007/s12029-024-01122-7

**Published:** 2024-10-16

**Authors:** Yousef Yahia, Ma’mon Qasem, Shahem Abbarh, Husam Saffo, Ibrahim M. Obeidat, Haidar Hussein Barjas, Mohanad Mohammed Faisal, Malik Halabiya, Prem Chandra, Moutaz Derbala

**Affiliations:** 1 Gastroenterology and Hepatology Department, Hamad Medical Corporation, Doha, Qatar; 2 Radiology Department, Hamad Medical Corporation, Doha, Qatar; 3 Internal Medicine Department, Hamad Medical Corporation, Doha, Qatar; 4 Medical Research Centre, Hamad Medical Corporation, Doha, Qatar

**Keywords:** Hepatocellular carcinoma, Liver cirrhosis, Liver nodules, Natural History

## Abstract

**Background:**

Several studies have shown a higher risk of liver cancer from indeterminate liver nodules, but the exact occurrence and predictors of liver cancer in this group are still unclear. Our aim is to study the development of liver cancer in this population and identify any potential risk factors.

**Methods:**

This retrospective study evaluated cirrhotic patients with indeterminate liver nodules from 2013 to 2023.Data from electronic patient records was analyzed to assess the association between HCC and baseline factors. Subgroup exploratory analysis compared characteristics of patients with de novo HCC and those with nodule transformation HCC.

**Results:**

Out of 116 patients with liver nodules, 19 (16%) developed HCC in up to 7.5-year follow-up. Univariate Cox regression analysis showed a significant association between HCC incidence and smoking [hazard ratio (HR) 2.60, 95% Confidence Interval [CI] 1.01–6.74), nodule diameter exceeding 2 cm (HR 5.41, 95% CI 1.45–20.18), and baseline LI-RADS score 3 (HR 3.78, 95% CI 1.36–19.52). Multivariate Cox regression analysis revealed significant independent associations with nodule diameters 1 cm to < 2 cm (adjusted HR 3.35, 95% CI 1.06–10.60) and greater than 2 cm (adjusted HR 5.85, 95% CI 1.10–31.16), as well as with LI-RADS 3 lesions (adjusted HR 3.75, 95% CI 1.16–12.11) with adjusting other potential predictors and covariates.

**Conclusion:**

Our findings show a higher incidence of HCC in patients with indeterminate liver nodules, increasing over time and reaching 30% at seven years. Nodules larger than 1–2 cm or LI-RADS 3 lesions pose increased risk for HCC. Enhanced surveillance is necessary given the lack of clear management guidelines.

**Supplementary Information:**

The online version contains supplementary material available at 10.1007/s12029-024-01122-7.

## Introduction

HCC is the fifth most common type of cancer worldwide [1] and is characterized by its rapid growth [2, 3]. It is also the leading cause of death among individuals with liver cirrhosis [4].Certain population studies suggest that HCC may become the third most common cause of cancer-related deaths in the USA [5, 6]. In the Gulf region, which is home to diverse expatriate communities, the age-standardized incidence rate (ASR) for HCC is 4.7 per 100,000 individuals, with a mortality rate of 4.5, as reported by the World Health Organization (WHO) [7]. In 2017, Qatar reported the highest liver cancer mortality rate among Gulf region countries [8]. Moreover, the incidence of HCC increases with age, with estimates indicating an annual rise of 1–8% [9]. The pathophysiology of HCC remains incompletely understood, but it is believed to entail a multi-step progression resulting in both micro and macroscopic alterations. These alterations give rise to preneoplastic nodules that require prompt and precise management. Additionally, HCC can develop independently of pre-existing indeterminate nodules, in a process known as de novo hepatocarcinogenesis [10, 11]. However, the extent to which indeterminate nodules alone increase the risk of HCC, either by originating from these nodules or arising de novo, has not been adequately studied.

In patients with cirrhosis, both American and European guidelines recommend screening for HCC every six months using abdominal ultrasound, as early detection can significantly improve outcomes [1, 12]. If suspicious lesions are found during the ultrasound, further evaluation should be conducted using contrast-enhanced abdominal computed tomography (CT) scan or magnetic resonance imaging (MRI) of the liver. The increased surveillance for HCC in clinical settings has led to a higher detection rate of indeterminate liver nodules. These liver nodules or lesions do not display the typical radiological features of HCC, such as arterial enhancement, venous phase washout, and delayed phase washout, nor do they exhibit any suspicious features that would warrant a liver biopsy and are usually labeled as Liver Imaging Reporting and Data System (LI-RADS) 2–3 lesions. Consequently, these indeterminant nodules present a challenge for clinicians as they lack the characteristic features of benign liver lesions, and, simultaneously, there are no clear guidelines for managing them [13]. Performing a liver biopsy for every indeterminate liver nodule is impractical due to the invasive nature of the procedure and the potential risks outweighing the benefits, particularly with the increasing number of nodules detected through radiological modalities. Although these nodules may not be cancerous, previous studies have indicated their potential to transform into HCC in the future [14, 15]. However, limited data is available on managing these nodules when found in cirrhotic patients during routine surveillance. Close observation has been the most common approach used, but there is still controversy over the imaging modality and the follow-up duration. For example, the American Association for the Study of Liver Disease (AASLD) practice guidance, published in 2018, suggests various options for indeterminate nodules, including follow-up imaging, imaging with a different modality or contrast agent, or biopsy. Nevertheless, the guidance does not recommend one option over the others [12].

It is difficult to assess the risk of HCC in patients with indeterminate liver lesions due to limited available data on the natural history of these lesions. The incidence of HCC in these patients varies widely, ranging from 14 to 65% [14–16]. In the past, research studies have typically overlooked cases of de novo hepatocellular carcinoma (HCC), focusing mainly on nodular outcomes without considering the potential occurrence of de novo HCC. To address this gap, we conducted an observational study to determine the overall incidence of HCC within this specific patient group, encompassing both de novo HCC and HCC arising from existing nodules. This approach could provide a new framework for exploring the impact of undetermined nodules on the surrounding liver tissue, thereby paving the way for further research. Additionally, the study aimed to identify potential predictors and radiological factors that contribute to the occurrence of HCC and to compare these findings with previous studies on HCC incidence in the general cirrhotic population and in cirrhotic patients with indeterminate liver nodules.

## Method

### Study Population and Design

We conducted a retrospective study at a single tertiary center, observing patients with cirrhosis who had at least one liver nodule that could not be definitively diagnosed as HCC between January 2013 and December 2023. These nodules are typically detected during routine chronic liver disease management at our hepatology clinic. Such nodules must not exhibit the characteristic radiological features of HCC, such as arterial enhancement, venous phase washout, delayed phase washout, or any suspicious features that necessitate a liver biopsy.

Our institution follows a specific protocol for monitoring cirrhotic patients with liver nodules. As part of this protocol, liver MRIs are regularly conducted for any liver nodule, and lesions are categorized using the Liver Imaging Reporting and Data System (LI-RADS) terminology. The LI-RADS categorizes liver lesions in high-risk patients with cirrhosis: LI-RADS-5 confirms HCC without the need for further histological diagnosis, whereas LI-RADS-1 indicates a benign lesion that does not require additional evaluation. The LI-RADS system is a detailed and comprehensive method of classifying liver lesions, and readers can find more information about it in the Supplementary Appendix. For liver nodules classified as LI-RADS-5 or LI-RADS-4, a multidisciplinary team comprising radiology, hepatology, oncology, interventional radiology, and hepatobiliary surgery reviews the case. For indeterminate nodules or those classified as LI-RADS-2 or LI-RADS-3, a repeat liver MRI is scheduled within 6 months. If the nodule increases in size or is reclassified as LI-RADS-4, it is referred to a multidisciplinary team for discussion and further evaluation. If a LI-RADS-2 nodule remains stable, it will be monitored with an abdominal ultrasound every 6 months. For LI-RADS-3 nodules, MRI surveillance will be conducted every 6 months for a period of 24 months. If the nodule remains radiologically stable during this period, follow-up with abdominal ultrasound will continue every six months.

Therefore, we identified our study population by searching the electronic medical records, including MRI reports, for specific terms such as "liver nodule," "hepatic nodule," "regenerative nodule," "dysplastic nodule," "indeterminate nodule," "LI-RADS-3," and "LI-RADS-2."

The start date was the date of the first MRI scan that reported an indeterminate lesion. Patients are usually monitored with MRI scans every 6 to 12 months. MRI images with these nodules are reviewed from the first MRI until the end of the study, lost to follow-up, or HCC development. Baseline characteristics of the patients (age, sex, etiology of liver disease, liver function tests, bilirubin, alpha-fetoprotein (AFP), Child–Pugh score, Model of End-stage Liver disease (MELD) score) and radiological features were collected from the electronic medical records. Experienced radiologists perform a comprehensive review of subsequent MRI scans to assess the progression of indeterminate lesions, track the transformation rate to HCC, and assign LI-RADS scores to the lesions. The diagnosis of HCC is determined based on the LI-RADS scoring system or, when necessary, histological diagnosis. Inclusion criteria included individuals aged 18 and older who have been diagnosed with cirrhosis and have at least one hepatic nodule detected by MRI. These nodules should not display definitive HCC (LI-RADS-5) or probable HCC (LI-RADS-4) features that require liver biopsy and pose a high risk of HCC. Additionally, the patient should have at least one stable MRI liver finding within 6–12 months from the initial MRI with the nodules. Exclusion criteria for the study included the absence of cirrhosis, definite HCC (LI-RADS-5) or probable HCC (LI-RADS-4) at baseline, and benign features that do not warrant further evaluation (LI-RADS-1), such as hemangioma. (Detailed eligibility criteria can be found in the Supplementary Appendix).

### Statistical Consideration and Data Analysis

For continuous variables, mean (SD) and median (IQR) were used for normally and non-normally distributed data, respectively. Categorical variables are displayed as frequencies and percentages. The study's primary outcome was time to HCC occurrence, and this was estimated using Kaplan–Meier curves. This was measured from the first MRI scan with liver nodules until the patient developed HCC, either de novo or on top of the previous nodule. In the case of censoring, the time was calculated until the last MRI scan before the end of the study. Associations between two or more qualitative data variables were assessed using Chi-square (χ2) test or Fisher Exact test as appropriate. Quantitative data between the two independent groups (HCC and non-HCC) were analyzed using unpaired t or Mann Whitney U test as appropriate. The evaluation of potential risk factors and predictors associated with HCC was studied using Cox regression models followed by Log-rank test to compare the incidence of HCC across various potential subgroups. The impact of clinical, biochemical, and radiological parameters on HCC was examined. Univariate Cox regression model was used to calculate hazard ratios (HR) and their 95% confidence intervals (CI) and to estimate the risk association between each covariate and HCC. After identifying the covariates significantly (considering both statistical and clinical significance) associated with HCC incidence, they were incorporated into a multivariate Cox regression model to establish independent associations. Furthermore, an exploratory statistical analysis was conducted for HCC patients, comparing the potential factors and radiological characteristics of the de novo HCC group and HCC on top of the nodule group.

A two-sided P-value < 0.05 was considered as statistically significant. All Statistical analyses were done using the statistical packages SPSS version 29.0 (Armonk, NY: IBM Corp) and Epi Info 2000 (Centers for Disease Control and Prevention, Atlanta, GA).

All methods followed the Declaration of Helsinki and the hospital's guidelines and regulations. The ethical committee waived the need for consent, as only anonymous data without patients' identifiers were provided to the research team. The study was approved by the ethics and research committee at the Medical Research Center of Hamad Medical Corporation, Doha, Qatar, with the approval number MRC-01–24-085.

## Results

We identified 116 patients who satisfied the inclusion criteria and presented with liver nodules. Among these individuals, 19 patients (16%) developed HCC; 6 had de novo HCC, and 13 had HCC originating from an indeterminate lesion. Table 1 presents the baseline demographic information, liver disease characteristics, and radiological features of the lesions at the time of the initial liver nodule detection. Most of the cohort were males (82%), and all HCC cases occurred in males (100%). The average age of the entire cohort was 50.17 (± 10.6) years. The primary ethnicity in both groups was Middle Eastern, followed by Asians. The cohort mainly consisted of non-smokers (73%) who had no history of alcohol consumption (87.9%), a pattern that was also observed in the group diagnosed with HCC.In the cohort, hepatitis C virus (HCV) infection was the main cause of liver cirrhosis, accounting for 50.9% of cases. It was even more prevalent in the HCC group, representing 87.9% of cases. No cases of HCC were attributed to metabolic-associated liver disease (MASLD). Child–Pugh grade A was the most common in both non-HCC and HCC groups. The majority of nodules (74.1%) were less than 1 cm in diameter, including 47% in the HCC group. The LI-RADS 2 score was present in 56% of the total cohort, 61.9% in the non-HCC group, and 26.3% in the HCC group.

**Table 1:** Demographic and clinical characteristics of included patients

Characteristics	All patients (n = 116)	No HCC Transformation (n = 97)	HCC Transformation (n = 19)	*P*-value
Age, mean (SD)	50.17 (± 10.6)	49.4 (± 11.0)	54 (± 6.7)	0.019
Sex: male, n (%)	96 (82.8)	77 (79)	19 (100)	**0.041**
Ethnicity, n (%)				0.32
Middle East	84 (72.4)	68 (70)	16 (84.2)	
Asian	28 (24.1)	26 (27)	2 (10.5)	
Others	4 (3.4)	3 (3)	1 (5.3)	
BMI, mean (SD)	29 (± 4.8)	29 (± 4.8)	29 (± 5.3)	0.066
Diabetes Mellitus: yes, n (%)	56 (48.3)	46 (47)	10 (52)	0.612
Smoking status, n (%)				0.069
Non-smoker	85 (73.3)	74 (76.3)	11 (57.9)	
Smoker	24 (20.7)	17 (17.5)	7 (36.8)	
Ex-smoker	0 (0.0)	0 (0.0)	0 (0.0)	
Missing	7 (6.0)	6 (6.2)	1 (5.3)	
Alcohol Intake, n (%)				0.068
No	102 (87.9)	87 (90)	15 (78.9)	
Yes	11 (9.5)	7 (7)	4 (21.1)	
Missing	3 (2.6)	3 (3)	0 (0.0)	
Etiology of Cirrhosis, n (%)				0.079
Alcohol	3 (2.6)	3 (3.1)	0 (0.0)	
NASH	17 (14.7)	17 (17.5)	0 (0.0)	
NASH and Alcohol	3 (2.6)	2 (2.1)	1 (5.3)	
HCV	59 (50.9)	44 (45.4)	15 (78.9)	
HCV and Alcohol	1 (0.9)	0 (0.0)	1 (5.3)	
HBV	15 (12.9)	13 (13.4)	2 (10.5)	
HBV and HCV	1 (0.9)	1 (1.0)	0 (0.0)	
Autoimmune	2 (1.7)	2 (2.1)	0 (0.0)	
PSC/PBC	3 (2.6)	3 (3.1)	0 (0.0)	
Cryptogenic	10 (8.6)	10 (10.3)	0 (0.0)	
Others	2 (1.7)	2 (2.1)	0 (0.0)	
Family History of HCC, n (%)				0.429
No	104 (89.7)	86 (89)	18 (94.7)	
Yes	3 (2.6)	3 (3)	0 (0.0)	
Missing	9 (7.8)	8 (8)	1 (5.3)	
Ascites, n (%)				0.629
No	93 (80.2)	77 (79)	16 (84)	
yes	23 (19.8)	20 (21)	3 (16)	
Child–Pugh Grade, n (%)				0.538
A	87 (75.0)	72 (74.2)	15 (78.9)	
B	23 (19.8)	19 (19.6)	4 (21.1)	
C	6 (5.2)	6 (6.2)	0 (0.0)	
Ongoing Liver Injury, n (%)				0.990
Yes	57 (49.1)	48 (49.5)	9 (47.4)	
No	57 (49.1)	48 (49.5)	9 (47.4)	
Missing	2 (1.7)	1 (1.0)	1 (5.3)	
ALT (unit/l), Median (IQR)	42.5 (23–64)	43 (23–65)	37 (21–77)	0.923
AST (unit/l), Median (IQR)	42.5 (26–70)	43 (26–71)	42 (26–70)	0.932
ALP (unit/l), Median (IQR)	93 (71–133)	93 (71–134)	93 (79–141)	0.961
Albumin (gram/l), mean (SD)	35 (± 6.80)	36 (± 7.0)	34 (± 6.16)	0.314
INR, mean (SD)	1.2 (± 0.24)	1.2 (± 0.24)	1.2 (± 0.15)	0.908
Platelet count (× 109/l), Median (IQR)	113 (77–146)	117 (84–151)	82 (58–138)	0.143
MELD score, Median (IQR)	8 (7–11)	8 (7–11)	8 (7–11)	0.873
AFP, Median (IQR)	5 (3–8)	5 (2.8–7)	7.5 (3.2–14)	0.064
Bilirubin (umol/l), median (IQR)	17 (12–26)	17 (12–27)	16 (13–26)	0.829
Radiological Features of Nodules				
Size of the Largest Nodule, n (%)				**0.007**
< 10 mm	86 (74.1)	77 (79.4)	9 (47.4)	
10-19 mm	24 (20.7)	17 (17.5)	7 (36.8)	
> = 20 mm	6 (5.2)	3 (3.1)	3 (15.8)	
LIRADS, n (%)				**0.004**
2	65 (56.0)	60 (61.9)	5 (26.3)	
3	51 (44.0)	37 (38.1)	14 (73.7)	
T2 signal, n (%)				**0.005**
Hypointense	26 (22.4)	26 (26.8)	0 (0)	
Iso-intense	84 (72.4)	68 (70.1)	16 (84.2)	
Hyperintense	6 (5.2)	3 (3.1)	3 (15.8)	
T1 signal, n (%)				0.092
Hypointense	0 (0)	0 (0)	0 (0)	
Iso-intense	36 (31.0)	27 (27.8)	9 (47.4)	
Hyperintense	80 (69.0)	70 (72.2)	10 (52.6)	
Arterial Enhancement: yes, n (%)	38 (32.8)	31 (32)	7 (36.8)	0.678
Delayed Washout: yes, n (%)	5 (4.3)	3 (3.1)	2 (10.5)	0.145

**Fig. 1:**
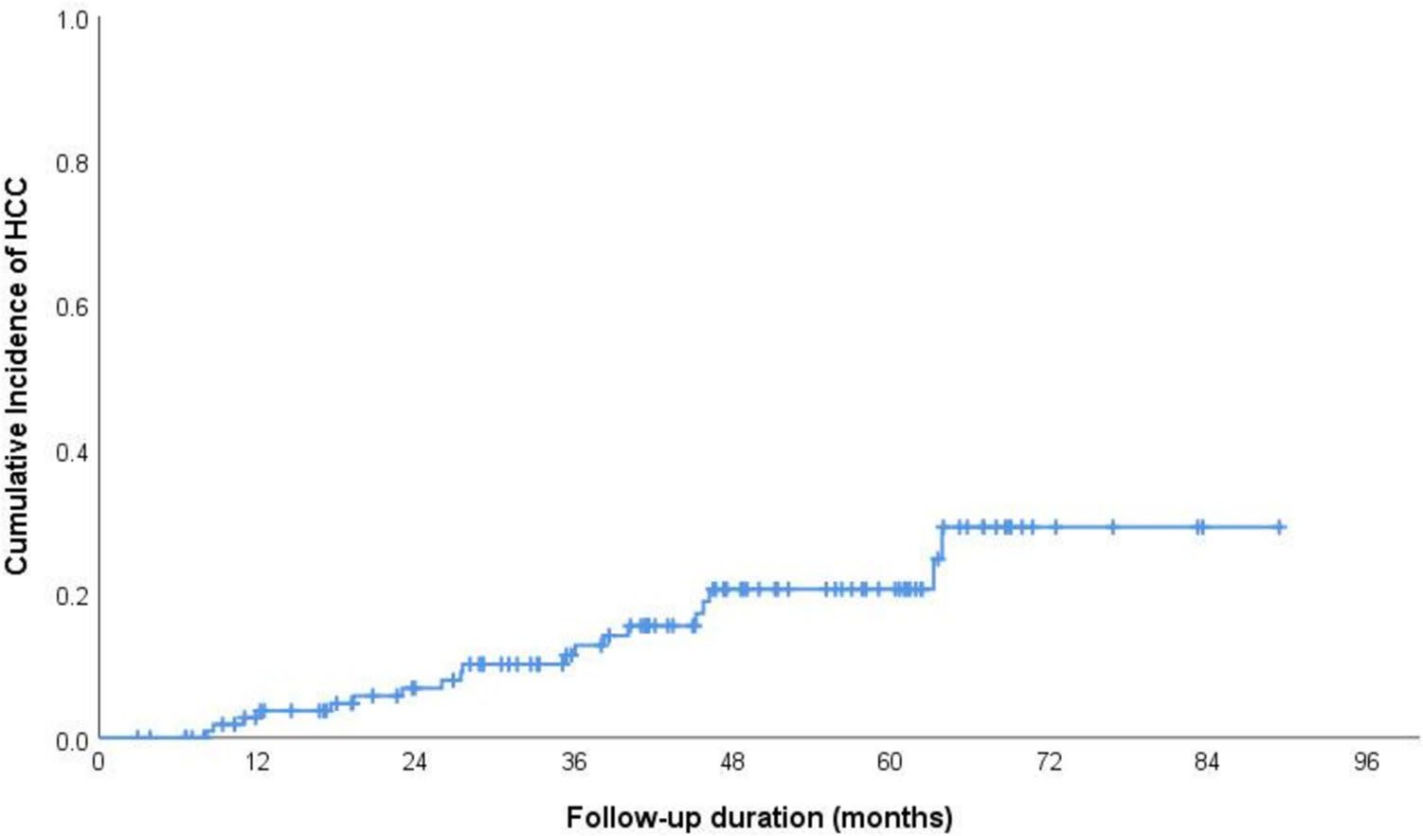
The cumulative incidence of HCC over follow up duration the cumulative incidence of HCC at 12, 36, 60, and 84 months was 3.7%, 10%, 20%, and 30%, respectively

**Fig. 2:**
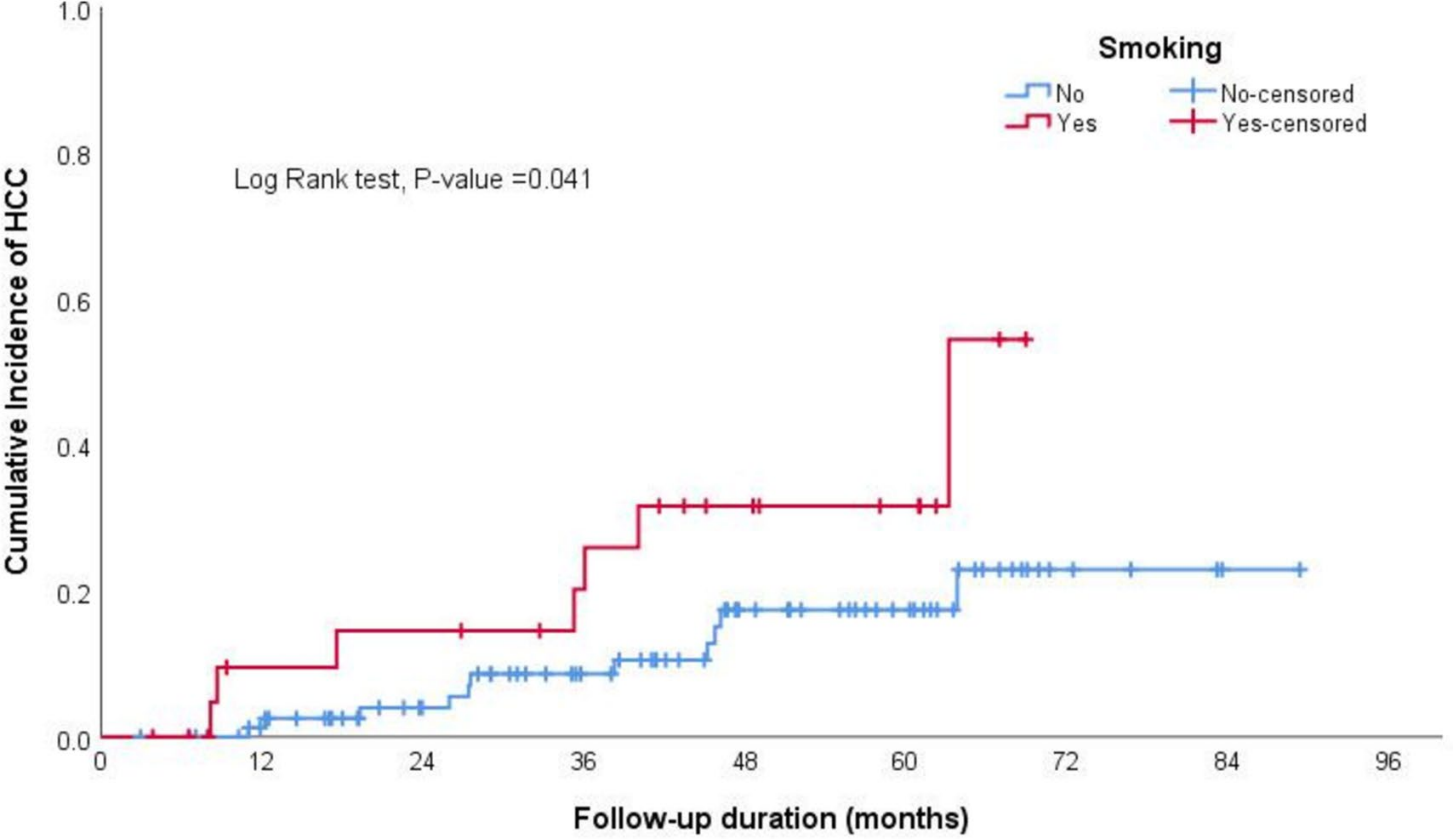
The log-rank test results from Kaplan–Meier estimates showed significantly higher cumulative incidence of HCC among smokers compared to non-smokers

**Fig. 3:**
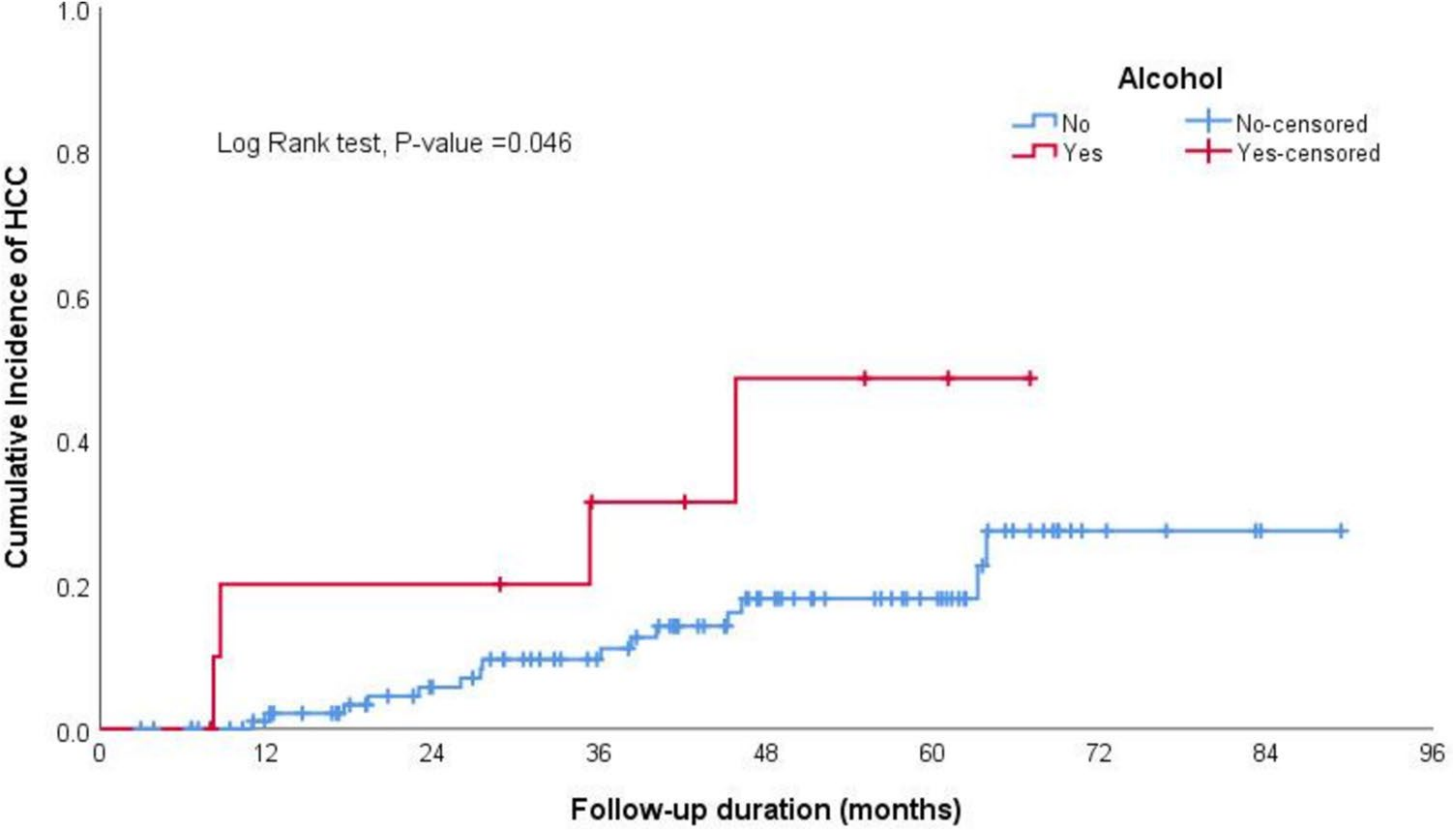
The log-rank test results from Kaplan–Meier estimates showed significantly higher cumulative incidence of HCC among alcohol intake than non-alcohol intake groups

**Fig. 4:**
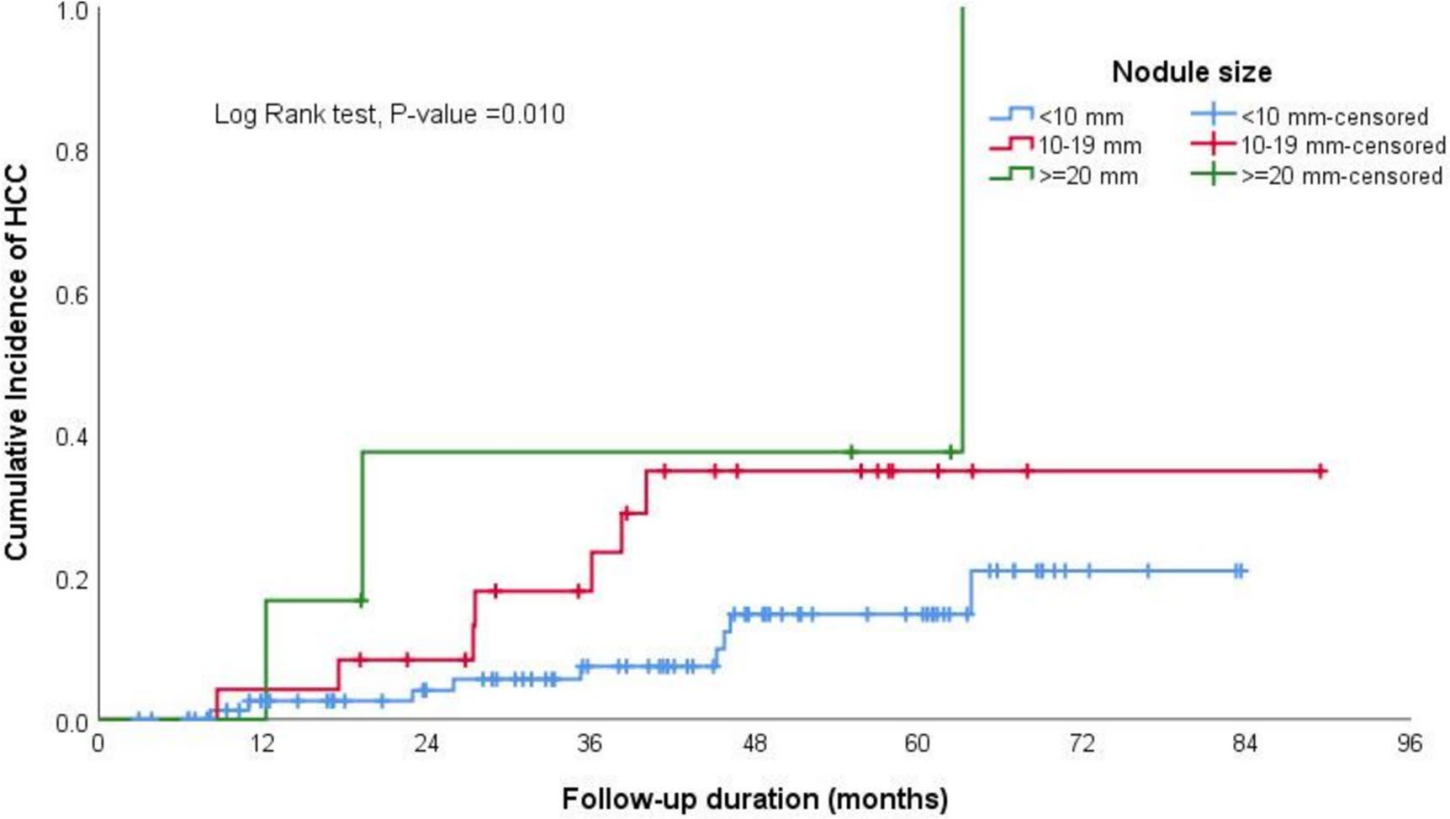
The log-rank test results from Kaplan–Meier estimates showed significantly higher cumulative incidence of HCC in patients with liver nodule size 1 to 2 cm and > 2 cm compared to nodule size < 1 cm

**Fig. 5:**
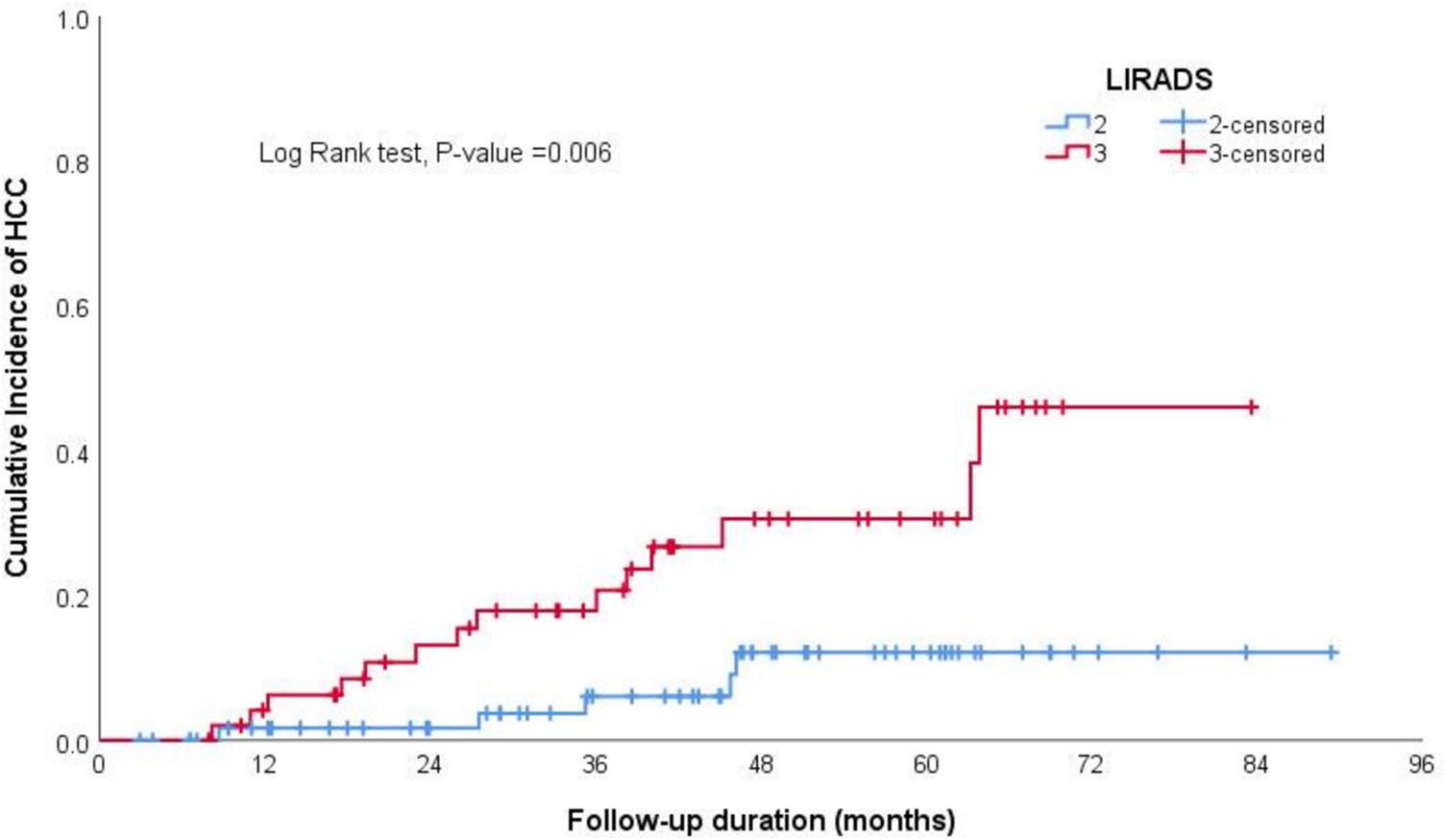
The log-rank test results from Kaplan–Meier estimates depicts significantly higher cumulative incidence of HCC in patients with LI-RADS-3 compared to LI-RADS-2

In the Kaplan–Meier curve analysis, the cumulative incidence of HCC at 12, 36, 60, and 84 months was 3.7%, 10%, 20%, and 30%, respectively, as illustrated in Fig. 1.The log-rank test results from Kaplan–Meier estimates indicated significant differences in incidence of HCC based on smoking status (non-smoker vs smoker), sex (male vs female), alcohol intake (history of alcohol intake—regardless of the amount vs no alcohol intake), size of the largest nodule at the time of diagnosis (less than 2 cm or above), and T2-signal by MRI (hyperintense or iso-hypointense) (Table 2, Figs. 2, 3, 4 and 5).

**Table 2:** Log rank test results from Kaplan–Meier estimates of survival

Covariate	Reference	Log Rank	*P-*value
Smoking	Non-smoker versus smoker	4.191	**0.041**
Sex (Male)	Male versus female	5.067	**0.024**
DM		0.755	0.385
Alcohol intake	History of alcohol intake versus no alcohol intake	3.987	**0.046**
Child Pugh Grade		0.601	0.741
Ongoing Liver Injury		0.063	0.802
Largest Nodule Diameter	Equal or Above 2 cm versus less than 2 cm	9.203	**0.010**
LI-RADS	LIRADS-3 versus LIRADS-2	7.516	**0.006**
T2 Signal (Hyperintense)	Hyperintense versus iso- or hypo-intense	9.941	**0.007**
T1 Signal	Hyperintense versus iso- or hypo-intense	2.558	0.110
Arterial Enhancement	Enhancement versus none	0.023	0.878
Delayed Washout	Presence versus absence of delayed washout	5.077	**0.024**

The univariate Cox regression analysis revealed a statistically significant association between the occurrence of HCC and smoking (hazard ratio [HR] 2.60, 95% Confidence Interval [CI] 1.01–6.74). Additionally, there was a notable association with baseline nodule diameter, remarkably when the size exceeded 2 cm (HR 5.41, 95% CI 1.45–20.18) and baseline LI-RADS 3 (HR 3.78, 95% CI 1.36–19.52) (Table 3).The multivariate Cox analysis revealed significant independent associations with nodule diameters greater than 1 cm (HR 3.35, 95% CI 1.06–10.60) and 2 cm (HR 5.85, 95% CI 1.10–31.16), as well as with LI-RADS 3 lesions (HR 3.75, 95% CI 1.16–12.11) (Table 4).

**Table 3:** Univariate Cox regression analysis

Covariates	Continuous/Categorical	Reference	Hazard Ratio (HR)	95% CI	*P-*value	Data Available (N)
Age at Diagnosis	Continuous		1.037	0.987–1.091	0.152	109
Sex	Categorical	Female	29.179	0.254–3353.285	0.163	109
BMI	Continuous		1.001	0.899–1.113	0.991	90
DM	Categorical	Non-DM	1.506	0.594–3.819	0.388	108
Smoking	Categorical	Non-smoker	2.603	1.006–6.736	**0.049**	102
Alcohol	Categorical	No hx of alcohol intake	2.929	0.969–8.851	0.057	106
ALT	Continuous		1.006	0.993–1.020	0.370	109
AST	Continuous		1.003	0.990–1.016	0.649	109
ALP	Continuous		0.999	0.991–1.006	0.717	109
Albumin	Continuous		0.940	0.871–1.014	0.110	109
Bilirubin	Continuous		1.003	0.987–1.019	0.751	109
Platelet Count	Continuous		0.994	0.984–1.004	0.232	107
MELD score	Continuous		1.043	0.935–1.163	0.454	108
AFP	Continuous		1.004	0.992–1.015	0.532	107
Largest Nodule Size	Categorical	< 10 mm	2.754	1.024–7.412	**0.045**	109
			5.405	1.448–20.177	**0.012**	
LIRADS	Categorical	2	3.784	1.361–10.521	**0.011**	109

**Table 4:** Multivariate Cox regression analysis

Covariates	Hazard ratio (HR)	95% CI	*P-*value
Smoking	1.216	0.393–3.767	0.735
Alcohol	3.288	0.75–14.417	0.114
Nodule size (10–19 mm)	3.347	1.057–10.603	**0.040**
Nodule size (≥ 20 mm)	5.854	1.10–31.159	**0.038**
LIRADS (3)	3.753	1.163–12.109	**0.027**

Subgroup exploratory analysis was conducted for the HCC group to descriptively compare the clinical, biochemical, and radiological features of patients with de novo HCC and patients with HCC arising from pre-existing nodules at the time of nodule diagnosis and HCC diagnosis (Table 5).The two subgroups had similar demographic features and blood test results, except for a higher platelet count in the de novo HCC group. The baseline radiological features showed differences, with more smaller nodules(< 1 cm) and LI-RADS2 predominance in the de novo HCC group. At the HCC time, there were no significant deviations from the initial baseline blood test results except for AFP levels in the de novo HCC group, which had a median of 50 (range 3.7–158) compared to a median of 9.5 (range 4.5–113) in the other group. Both groups showed a consistent increase in the size of the primary nodule at the time of HCC diagnosis, with 50% of the nodules in the de novo cohort being 2 cm or larger, compared to 62% in the other group.

**Table 5:** Demographic and clinical characteristics of included patients

Characteristics of HCC patients	Denovo HCC (*n* = 6)	HCC from Preexisting Nodule (*n* = 13)
Age, mean (SD)	56 (± 5.24)	53 (± 7.33)
Smoking status, n (%)		
Non-smoker	3 (50.0)	8 (61.5)
Smoker	3 (50.0)	4 (30.8)
Etiology of Cirrhosis, n (%)		
Alcohol	0 (0)	0 (0)
NASH	0 (0)	0 (0)
NASH and Alcohol	1 (16.7)	0 (0)
HCV	5 (83.3)	10 (76.9)
HCV and Alcohol	0 (0)	1 (7.7)
HBV	0 (0)	2 (15.4)
HBV and HCV	0 (0)	0 (0)
Autoimmune	1 (0)	0 (0)
PSC/PBC	2 (0)	0 (0)
Cryptogenic	3 (0)	0 (0)
Others	4 (0)	0 (0)
Child–Pugh Grade, n (%)		
A	4 (66.7)	11 (84.6)
B	2 (33.3)	2 (15.4)
Baseline Results:		
ALT (unit/l), Median (IQR)	34 (25–51)	48 (21–82)
AST (unit/l), Median (IQR)	46 (41–55)	42 (24–79)
ALP (unit/l), Median (IQR)	80 (70–81)	105 (90–148)
Albumin (gram/l), mean (SD)	32 (± 7.6)	35 (± 5.0)
Bilirubin (umol/l), median (IQR)	29 (26–64)	13 (11.5–17.5)
Platelet count (× 109/l),Median (IQR)	138 (51–159)	81 (63–124)
MELD score, Median (IQR)	10.5 (10–13)	7 (7–9)
AFP, Median (IQR)	6 (3.2–7.6)	9 (3.5–16.5)
Radiological Features of Nodules		
Size of the Largest Nodule, n (%)		
< 10 mm	4 (66.7)	5 (38.5)
10-19 mm	2 (33.3)	5 (38.5)
> = 20 mm	0 (0)	3 (23.1)
LIRADS, n (%)		
2	4 (66.7)	1 (7.7)
3	2 (33.3)	12 (92.3)
T2 signal, n (%)		
Hypointense	0 (0)	0 (0)
Iso-intense	6 (100)	10 (76.9)
Hyperintense	0 (0)	3 (23.1)
T1 signal, n (%)		
Hypointense	0 (0)	
Iso-intense	2 (33.3)	7 (53.8)
Hyperintense	4 (66.7)	6 (46.2)
Arterial Enhancement: yes, n (%)	1 (16.7)	6 (46.2)
Delayed Washout: yes, n (%)	0 (0)	2 (15.4)
Capsular Enhancement: yes, n(%)	0 (0)	0 (0)
HCC time (event) Results:		
ALT (unit/l), Median (IQR)	30 (24–56)	25 (18.5–40)
AST (unit/l), Median (IQR)	38 (30–70)	28 (21–45.5)
ALP (unit/l), Median (IQR)	105 (86–119)	90 (60–119)
Albumin (gram/l), mean (SD)	29 (± 5.8)	37 (± 6.5)
Bilirubin (umol/l), median (IQR)	25 (23–30)	14 (11.5–21)
Platelet count (× 109/l), Median (IQR)	97 (72–100)	118 (88–155)
MELD score, Median (IQR)	10.5 (10–11)	8 (7–11)
AFP, Median (IQR)	50 (3.7–158)	9.5 (4.5–113)
Child–Pugh Grade, n (%)		
A	4 (66.7)	10 (76.9)
B	2 (33.3)	2 (15.4)
C	0 (0)	1 (7.7)
Radiological Features of Nodules at HCC time		
Size of the Largest Nodule, n (%)		
< 10 mm	0 (0)	0 (0)
10-19 mm	3 (50)	5 (38)
> = 20 mm	3 (50)	8 (62)

## Discussion

This is a real-world data study; our primary objective was to evaluate the risk of HCC in patients with indeterminate liver nodules. Our findings revealed that 16% of the patients developed HCC during the study period, with cumulative incidence rates at 1, 3, 5, and 7 years of 3.7%, 10%, 20%, and 30%, respectively. Comparing our results with previous studies that evaluated the cumulative incidence of HCC in cirrhotic patients, we observed that our cohort exhibited a higher cumulative incidence of HCC over the years. For instance, a Swedish nationwide retrospective study found a cumulative incidence of HCC in cirrhosis of 8.3% at 5 years [17]. Ioannou et al. found a 4.7% HCC incidence over 3.6 years [9], while Paranaguá Vezozzo DC et al. reported a 14.3% cumulative incidence over 5 years [18]. We also compared our results with studies that assessed the risk of HCC transformation from preexisting indeterminate nodules. Cococcia et al., in a retrospective study of 109 patients with indeterminate nodules, found that HCC developed in one-fifth of the cases over 4.6 years, which closely aligns with our own [14]. Another study indicated that the 3-year rate of nodule transformation to HCC was 15.7% [15]. Kobayashi et al. reported cumulative HCC development rates of 3.3%, 9.7%, and 12.4% in the first, third, and fifth years, respectively, for regenerative nodules [19]. Beal et al. followed patients with indeterminate lesions and reported an HCC incidence of 21% in 4 years, consistent with our findings [20]. Although there is some variation in HCC incidence among these studies, the overall trend indicates a progressive increase in HCC incidence over time, particularly during the five-year period, with a more significant rise in patients with these nodules. Several theories have been proposed to explain the rising incidence of hepatocellular carcinoma (HCC) in the context of liver cirrhosis over time. These include the potential influence of chronic hepatic inflammation, promoted hepatocyte DNA synthesis, and the accrual of genomic alterations over time [21].

In our cohort, nearly 88% of HCC cases were attributed to HCV-related cirrhosis, consistent with findings from other studies [14, 15, 22]. The increased incidence of HCC in individuals with HCV may stem from the direct carcinogenic effects of specific HCV viral proteins involved in various oncogenic processes. Additionally, differences in the pathogenesis of necroinflammation between HCV and other etiologies, such as MASLD and alcohol-related liver disease, may lead to varying risks of HCC development [14, 22]. Our study revealed a higher incidence of HCC in males compared to females, likely influenced by demographic, behavioral, and environmental factors, consistent with findings from other epidemiological studies [14, 23]. In our univariate analysis, we found associations between smoking, alcohol intake, and male gender as predictors of HCC, consistent with previous studies [14, 23, 24]. However, these associations did not remain significant in the multi-regression model. Additionally, our multivariate regression analysis revealed that nodule size, especially nodules larger than 1-2 cm and LI-RADS-3 nodules, were independently associated with HCC, the same findings seen in previous studies. For instance, Khalili et al. reported a malignancy rate of 14%-23% for indeterminate 1–2-cm nodules[16].Similarly, Cococcia et al. found a higher incidence of HCC in patients with lesions ≥ 1 cm. However, the author did not find a significant association between lesion size and HCC in regression analysis, possibly due to the small sample size [14].

It is believed that the higher risk of hepatocellular carcinoma (HCC) associated with larger nodular size (especially > 1 cm) may be attributed to a potentially higher growth rate and a shorter tumor volume-doubling time, which are linked to the progression of nodules to hypervascular HCC [25]. In a meta-analysis of 12 studies evaluating LI-RADS-3 lesions, the risk of developing HCC ranged from 15.8% to 44.4% [26]. The presence of hyperintense T2 lesions may potentially indicate the development of HCC. However, our multi-regression model analysis did not reveal any significant correlations. These results are consistent with those reported by Rimola et al. [27].

Our research found that Child–Pugh grade, MELD score, and platelet count level were not predictors for HCC transformation despite their previously established correlation in studies [14, 15]. Our analysis indicated that the majority of cases at baseline and at HCC time were classified as Child–Pugh grade A or B rather than C, highlighting the limitations of relying on Child–Pugh grade for HCC prediction. These findings align with a retrospective study of HCC patients, where 78.7% of the patients were identified as Child–Pugh A or B [28]. During the follow-up period, two cases exhibited nodule disappearance. Additionally, there were five mortality cases during the study period, all of which were in the non-HCC group, with three attributed to complications related to liver disease. In the subgroup exploratory analysis for the HCC group, the study compared the characteristics between patients with denovo HCC and those with nodule transformation HCC. It's important to note that the study was purely descriptive and did not conduct any inferential analysis. Therefore, the findings may not be generalizable. However, the primary aim was to identify distinct features between both groups, as detailed in the results section. It's worth mentioning that denovo HCC occurred in 32% of all HCC cases, aligning with findings from Arvind et al. These findings indicate the possibility of inflammatory and covertly carcinogenic conditions that could increase the vulnerability of the entire liver to the development of HCC [29].

In previous studies on indeterminate nodules, denovo cases were typically excluded as the focus was solely on the nodules' outcome. In our study, however, we sought to assess the overall incidence of HCC in these populations. This approach makes our results more applicable, as patients and clinicians are concerned with the overall incidence of HCC, irrespective of whether the tumor originated from previous nodules. Our study has several strengths. These include a relatively large sample size, precise criteria for nodule diagnoses, the use of multiple MRI keywords during patient search to reduce selection bias, a lengthy follow-up period (with the majority of cases being followed up for 3–5 years and almost 25% observed for up to 7.5 years), a diverse patient population in terms of ethnicity at our center, and comprehensive radiological descriptions of the nodules provided by a specialized radiologist. However, the study has some limitations, mainly due to its retrospective design, which introduces potential biases such as observer bias and hidden unmeasured confounders. The data collection process relied on records, occasionally leading to incomplete information. As the field of artificial intelligence (AI) progresses in the realm of medicine, it is imperative that further research be conducted using deep learning AI and incorporating multiomics, such as radiomics and genomics. This will provide a more comprehensive understanding of the disease processes and aid in predicting the risk of HCC transformation from indeterminate liver nodules. Controlled prospective studies should be conducted over an extended period to establish the risk of HCC in patients with indeterminate liver nodules. Additionally, these studies can explore the potential of a carcinogenic microenvironment that may elevate the risk of metachronous HCC outside the nodules. By embracing a comprehensive approach, a more accurate risk assessment and an improved understanding of potential relationships can be achieved.

## Conclusion

The incidence of hepatocellular carcinoma is significantly high in patients with indeterminate liver nodules and tends to increase over time, with a cumulative incidence of 30% at seven years. Patients with nodules larger than 1–2 cm or LI-RADS 3 lesions are at a heightened risk of developing HCC. Additionally, the incidence of HCC is higher in HCV-related cirrhosis. These findings emphasize the need for increased surveillance in this population, particularly given the lack of established consensus on management. Thus, it is evident that there is a pressing requirement for prospective studies that incorporate artificial intelligence through the utilization of deep learning methodologies to establish the optimal strategy for this particular patient population.

## Supplementary Information

Below is the link to the electronic supplementary material.

Supplementary file1 (DOCX 16 KB)

## Data Availability

No datasets were generated or analysed during the current study.

## Abbreviations

*AASLD*: American Association for the Study of Liver Disease
*AI*: Artificial intelligence
*CI*: Confidence Interval
*CT*: Computed Tomography
*HCC*: Hepatocellular Carcinoma
*HCV*: Hepatitis C Virus
*HR*: Hazard Ratio
*LI-RADS*: Liver Imaging Reporting and Data System
*MASLD*: Metabolic-Associated Liver Disease
*MELD*: Model of End-stage Liver disease
*MRI*: Magnetic Resonance Imaging
*USA*: United States of America
*WHO*: World Health Organization
