# Supplementary material for: Risk of Malignancy in Indeterminate Liver Nodules Among Patients with Cirrhosis: A Retrospective Cohort Study
Source: J Gastrointest Cancer. 2024 Oct 16;56(1):1. doi: 10.1007/s12029-024-01122-7 (PMC11485135; doi:10.1007/s12029-024-01122-7)
Supplement: Supplementary file 1 — Supplementary file1 (DOCX 16 KB) [file 12029_2024_1122_MOESM1_ESM.docx]

**Supplementary Appendix**

**Inclusion Criteria:**

- Age over 18 years.

- Both genders are eligible.

- A documented history of liver cirrhosis from any cause.

- The presence of at least one hepatic nodule by MRI that does not have definite HCC (LIRADS-5) or probable HCC (LIRADS-4) features.

- At least one stable MRI liver finding within 6-12 months from the first MRI with the nodules.

- Liver nodules follow-up modality by MRI only.

- At least one-year follow-up from the first MRI with hepatic nodules.

**Exclusion criteria:**

- Absence of liver cirrhosis.

- Definite HCC (LIRADS-5) or probable HCC (LIRADS-4) at baseline.

- Features of metastatic liver lesions.

- Liver transplant as a baseline.

- Lesions with benign features (LIRADS-1) such as hemangioma.

- Insufficient follow-up of the nodule less than 6-12 months from initial detection.

**The LI-RADS score system:**

**LR-1 (100% benign)**

- Imaging features diagnostic of a benign entity:
  - cyst
  - hemangioma
  - vascular anomaly
  - perfusion alteration
  - focal scar

**LR-2 (probably benign)**

- Entities are similar to LR1, but the appearance is highly suggestive of the entity instead of 100% diagnostically confident.

**LR-3 (intermediate probability for HCC)**

- not a definitely benign entity, but not definitely HCC
- Includes entities with the following features:
  - not a definite mass
  - mass with hepatic arterial phase iso- or hypoenhancement
    - <20 mm with *no more than one*of the following:
      - non-peripheral "washout"
      - capsule
      - threshold growth
  - mass with hepatic arterial phase hyperenhancement
    - <20 mm with no "washout," capsule, or threshold growth

**LR-4 (probably HCC)**

- no arterial phase hyperenhancement
  - <20 mm
    - *two or more* of the following
      - non-peripheral "washout"
      - enhancing capsule
      - threshold growth
  - ≥20 mm
    - *one or more*of the following
      - non-peripheral "washout"
      - enhancing capsule
      - threshold growth
- **non-rim arterial phase hyperenhancement**
  - <10 mm
    - *one or more*of the following
      - non-peripheral "washout"
      - enhancing capsule
      - threshold growth
  - 10-19 mm
    - Enhancing "capsule" but does not meet threshold growth or washout criteria
  - ≥20 mm
    - *No*major suspicious features:
      - non-peripheral "washout"
      - enhancing capsule
      - threshold growth

**LR-5 (100% definite HCC)**

- **non-rim arterial phase hyperenhancement**
  - 10-19 mm
    - *single*major suspicious feature (washout or threshold growth), excluding enhancing "capsule" (LR-4)
    - *two or more* of the following
      - threshold growth
      - enhancing capsule
      - non-peripheral "washout"
  - ≥20 mm
    - ​*one or more* of the following
      - threshold growth
      - enhancing capsule
      - non-peripheral "washout"
